# Rapid Molecular Characterization of *Acinetobacter baumannii* Clones with rep-PCR and Evaluation of Carbapenemase Genes by New Multiplex PCR in Hospital District of Helsinki and Uusimaa

**DOI:** 10.1371/journal.pone.0085854

**Published:** 2014-01-21

**Authors:** Tanja Pasanen, Suvi Koskela, Sointu Mero, Eveliina Tarkka, Päivi Tissari, Martti Vaara, Juha Kirveskari

**Affiliations:** Division of Clinical Microbiology, Helsinki University Hospital, Helsinki, Finland; St. Petersburg Pasteur Institute, Russian Federation

## Abstract

Multidrug-resistant *Acinetobacter baumannii* (MDRAB) is an increasing problem worldwide. Prevalence of carbapenem resistance in *Acinetobacter* spp. due to acquired carbapenemase genes is not known in Finland. The purpose of this study was to examine prevalence and clonal spread of multiresistant *A. baumannii* group species, and their carbapenemase genes. A total of 55 *Acinetobacter* isolates were evaluated with repetitive PCR (DiversiLab) to analyse clonality of isolates, in conjunction with antimicrobial susceptibility profile for ampicillin/sulbactam, colistin, imipenem, meropenem, rifampicin and tigecycline. In addition, a new real-time PCR assay, detecting most clinically important carbapenemase genes just in two multiplex reactions, was developed. The assay detects genes for KPC, VIM, IMP, GES-1/-10, OXA-48, NDM, GIM-1, SPM-1, IMI/NMC-A, SME, CMY-10, SFC-1, SIM-1, OXA-23-like, OXA-24/40-like, OXA-58 and IS*Aba*I-OXA-51-like junction, and allows confident detection of isolates harbouring acquired carbapenemase genes. There was a time-dependent, clonal spread of multiresistant *A. baumannii* strongly correlating with carbapenamase gene profile, at least in this geographically restricted study material. The new carbapenemase screening assay was able to detect all the genes correctly suggesting it might be suitable for epidemiologic screening purposes in clinical laboratories.

## Introduction

*Acinetobacter baumannii* is a hospital-acquired pathogen which commonly causes pneumonia, bloodstream infections, meningitis, wound infections and urinary tract infections, especially in patients with impaired host defences. *A. baumannii* isolates are resistant to many antimicrobial classes: fluoroqinolones, tetracyclines, cephalosporines and aminoglycosides [1]. However, today carbapenem resistance is more frequently encountered [1]–[3]. In *A. baumannii* carbapenem resistance is usually conferred by carbapenem-hydrolyzing class D oxacillinases (CHDLs), including OXA-23-like (*bla*_OXA-23-like_), OXA-40-like (*bla*_OXA-40-like_), OXA-58-like (*bla*_OXA-58-like_), and OXA-143-like (*bla*_OXA-143-like_) oxacillinases. Additionally *A. baumannii* has the intrinsic OXA-51-like (*bla*_OXA-51-like_) oxacillinase [4], [5]. Although CHDLs exhibit weak carbapenem hydrolysis, they can confer resistance when overexpressed. This resistance is mediated through a combination of naturally low permeability to β-lactams, efflux pumps and IS*Aba* elements located upstream of the gene, providing a strong promoter activity [6]. In addition, *A. baumannii* may harbour many other carbapenemases more commonly found among *Enterobacteriaceae* and *Pseudomonas* species [7].

To determine genetic and epidemiological relatedness, genomic fingerprinting of clinical isolates is required. One of the most effective method is the repetitive extragenic palindromic sequence-based polymerase chain reaction (rep-PCR), which is commercially available known as the DiversiLab microbial typing system (bioMérieux, Marcy L'Etoile, France) [8]. This system has been proven useful in the typing of *A. baumannii* and has demonstrated good discriminatory ability, comparable with pulsed-field gel electroproresis (PFGE) and multilocus sequence typing (MLST) [9], [10]. Recently this rep-PCR typing system, DiversiLab, has identified eight carbapenem-resistant *A. baumannii* clonal lineages (WW1 to WW8) that are distributed worldwide [4]. DiversiLab fingerprints between laboratories were recently tested and clustering was found to be conserved [11].

The carbapenem resistance has recently attracted new interest as a subset among tens of gene families has spread to *Enterobacteriaceae* [12]–[14], despite a much longer history among *Pseudomonas* and *Acinetobacter* species. *A. baumannii* may harbour most of the acquired carbapenemase genes within *Enterobacteriaceae*, and *Pseudomonas* in addition to their characteristics CDHL genes [7].

Recently, new molecular assays have been described to detect most prevalent carbapenemase genes [15], or a subset of *A. baumannii* selective carbapenemase genes. Due to limited gene set, or technical limitations, most new tests are not suitable for clinical routine monitoring in low prevalence settings [16]. In addition, combinations of other resistance mechanisms, such as reduced permeability due porin mutations, or defect, and efflux pumps in conjunction with ampC β-lactamases are the most common cause of carbapenem resistance in low prevalence areas [14]. Therefore, an imipenem hydrolysis test or dedicated MALDI-TOF [17] and more extensive screening of resistance mechanisms in a reference laboratory are often needed to reliably exclude carbapenemase genes.

The aim of this study was to investigate the carbapenemase genes of *A. baumannii* and the correlation between these genes and clonal lineages. The feasibility of a new real-time PCR assay was tested for screening of most important carbapenemase genes detected among *A. baumannii*, *Enterobacteriaceae*, and *Pseudomonas* species.

## Materials and Methods

### Bacterial strains and culture conditions

A total of 55 *Acinetobacter* isolates from 44 patients were detected. 51 isolates with reduced susceptibility to carbapenem from HUSLAB (Laboratory of Helsinki University Central Hospital) between Jun 18^th^ 1993 and Jan 18^th^ 2008 were collected and four *Acinetobacter* isolates suscebtible to carbapenems were included as controls. Helsinki University Hospital is responsible for the secondary and tertiary care of app. 1.5 million people. The culture samples from this area received by HUSLAB are both from these hospitals as well as from outpatients of this geographical area, the Helsinki and Uusimaa district in southern Finland. The culture samples in this study were from patients treated in nine different hospitals (Table S1).

*Acinetobacter* isolates were cultured in aerobic atmosphere on chocolate and cysteine lactose electrolyte deficient (CLED) agar and incubated at 35°C for 18 h. Colonies with typical morphology and biochemistry were identified as *A. baumannii* complex. Identification with the VITEK 2 (bioMérieux, Marcy L'Etoile, France) system with GN card was performed, as well. 16S rRNA gene sequencing was performed when biochemical identification was equivocal. In addition a house-keeping OXA-51-like (*bla*_OXA-51-like_) gene was detected separately within all the clinical isolates with reduced susceptibility to carbapenems, whereas carbapenem susceptible control strains did not harbour OXA-51-like genes.

Antimicrobial susceptibility testing was performed by the disk diffusion method according to the CLSI guidelines (http://www.clsi.org). MICs for ampicillin/sulbactam, colistin, imipenem, meropenem, rifampicin and tigecycline by E-test (AB BIODISC, Solna, Sweden) were determined on Mueller-Hinton agar according to manufacturer's instructions.

### Design of multiplex Real-Time carbapenemase gene screening assay

The assay was designed to detect most clinically relevant carbapenemase genes described within *A. baumannii*, *Pseudomonas aeruginosa*, and *Enterobacteriaceae* species. The design was performed using AlleleID software (http://www.premierbiosoft.com), taking into account all the globally known sub-variants in NCBI data base. For practical purposes, the assay was divided in two multiplex reactions consisting of nine and eight gene families, respectively. The assay was validated *in vitro* using 43 positive control strains (Table 1), which were confirmed at National Institute for Health and Welfare, Turku, Finland [14]. Since the target primer regions were fully conserved in silico, it was considered adequate to demonstrate PCR performance with one or more control species representing all the gene variants. In addition, synthetic gene constructs for SFC, CMY-1/10, SIM, SME, OXA-25, and OXA-58 genes containing a partial, non-functional resistance gene in *E. coli* plasmid (pIDTsmart), including the amplicon and app. 20 bp upstream and downstream sequence (Integrated DNA Technologies Inc, CA, USA). The plasmid was then transfected into the TOP10 strain according to manufacturer's instructions. The construct was ordered from IDT using pSMART plasmid, blunt-ended, containing a kanamycin resistance gene. The SFC, and SIM the control strains were obtained later (as a kind gift from Dr. Correia and Dr. Yunsop Chong and Kyungwon Lee, consequently). All the gene products were confirmed by sequencing with reference primers, or the gene specific primers alone, when published reference primers were not available. For additional species identification, OXA-51 gene (*bla*_OXA-51-like_), with or without IS*AbaI*, was detected separately, using F_oxa51_001 AATTTATTTAACGAAGCACACACTACGG, and R_oxa51_001 GCACGAGCAAGATCATTACCATAGC primers and the PCR program shown below.

**Table 1:** Description of validation isolates.

Target	Species	Isolation site	Travel history
GES-1	P. aeruginosa	wound	no
GES-14	K. pneumoniae	trachea	n/a
GES-5	P. aeruginosa	incision wound	no
GES-5	P. aeruginosa	incision wound	no
IMI-1	E. cloacae	stool	Thailand
IMI-2	E.cloacae	wound	no
IMP-15	P. aeruginosa	blood	no
IMP-15	P. aeruginosa	wound	no
IMP-15	P. aeruginosa	incision wound	no
IMP-15	P. aeruginosa	urine	no
IMP-15	P. aeruginosa	urine	n/a
IMP-15	P. aeruginosa	incision wound	n/a
IMP-15	P. aeruginosa	urine	n/a
ISAbaI-OXA-51	A. baumannii	stool	Spain
ISAbaI-OXA-51	A. baumannii	stool	no
ISAbaI-OXA-51	A. baumannii	trachea	no
KPC	K. pneumoniae	stool	US
KPC-2	K. pneumoniae	stool	Greece
KPC-2	K. pneumoniae	wound	Italy
KPC-2	K. pneumoniae	urine	no
KPC-2	K. pneumoniae	blood	Mexico/US
KPC-2	K. pneumoniae	urine	no
NDM-1	K. pneumoniae	stool	n/a
OXA-23	A. baumannii	blood	n/a
OXA-23	A. baumannii	wound	no
OXA-23	A. baumannii	trachea	Thailand
OXA-48	E. coli	stool	Syria
OXA-48	K. pneumoniae	stool	Turkey
OXA-48	A. baumannii	stool	n/a
OXA-58	A. baumannii	stool	Tunis
OXA-58	A. baumannii	wound	no
OXA-58	A. baumannii	wound	no
OXA-58	A. baumannii	stool	Greece
OXA-58	A. baumannii	incision wound	n/a
OXA-58	A. baumannii	urine	no
SFC-1	S. fonticola	control strain	Portugal
SIM-1	A. baumannii	control strain	South-Korea
SME	S. marcescens	control strain	n/a
VIM	P. aeruginosa	stool	Thailand
VIM	K. pneumoniae	stool	Spain
VIM	K. pneumoniae	stool	Greece
VIM	K. pneumoniae	CV cathether	n/a
VIM-1	K. pneumoniae	blood	Greece
VIM-2	P. aeruginosa	trachea	Russia
VIM-2	P. aeruginosa	trachea	Russia

The specificity was tested with 58 carbapenem susceptible *Enterobacteriaceae* isolates (Table S2) [18], and 710 isolates with putative reduced susceptibility *A. baumannii*, *P. aeruginosa* and *Enterobacteriaceae* isolated from clinical samples during 2008–2011. These isolates were selected among samples growing on CHROMagar ESBL, or CHROMagar KPC plates (bioMérieux, Marcy L'Etoile, France), or from other culture isolates with disk diffusion diameter <25 mm for ertapenem, or <22 mm for meropenem, or MIC>0,5 mg/l for ertapenem and meropenem.

### Validation of multiplex Real-Time PCR assay

Template DNA was extracted from a single colony on CLED plate grown overnight, and re-suspended in 100 µl TE-buffer (0,5 McF) and boiled 15 min. Each 20 µl real time PCR-reaction included 10 µl Maxima SYBR Green qPCR Master Mix (2X) (Scientific Fermentas, Schwerte, Germany), 6 µl Oligomix 1 or 2 (Table 2), IDT (Integrated DNA Technologies, Inc.), 3 µl H_2_0, and 1 µl DNA template. Amplification was performed as follows: 95°C 10 min initial denaturation, 30 cycles with 95°C 20 sec denaturation, 58°C 30 sec annealing and extension, final extension 58°C 1 min and final denaturation 95°C 30 sec (MxPro 3005P, Stratagene, La Jolla, CA, USA). Melting curve was determined between temperatures 58–95°C. Control strains are presented in Table 3.

**Table 2:** Primers used for amplification of resistance genes by polymerase chain reaction (PCR).

Primer	Sequence 5′- 3′	Reference	Oligomix
F_ges_001	ACACCTGGCGACCTCAGAGATAC	This study	1
R_ges_001	ACTTGACCGACAGAGGCAACTAATTC	This study	1
F_gim_001	CGAATGGGTTGGTAGTTCTGGATAATAATC	This study	1
R_gim_001	ATGTGTATGTAGGAATTGACTTTGAATTTAGC	This study	1
F_imi1_001	AAACAAGGGAATGGGTGGAGACTG	This study	1
R_imi1_001	AAGGTATGCTTTGAATTTGCGTTG	This study	1
F_imp_10	AATAATGACGCCTATCTAATTGACACTCC	This study	1
R_imp_10	ATTCCACCCGTACTGTCGCTATG	This study	1
F_imp_11	TGACGCCTATCTGATTGACACTCC	This study	1
R_imp_11	GCTGTCGCTATGGAAATGTGAGG	This study	1
F_kpc_001	CAGCGGCAGCAGTTTGTTGATTG	This study	1
R_kpc_001	CCAGACGACGGCATAGTCATTTG	This study	1
F_oxa48_003	TTACTGAACATAAATCACAGGGCGTAG	This study	1
R_oxa48_003	ATTATTCGTAAATCCTTGCTGCTTATTCTC	This study	1
F_sme_006	CAGATGAGCGGTTCCCTTTATGC	This study	1
R_sme_006	CAGAAGCCATATCACCTAATGTCATACC	This study	1
F_spm_001	CCTACAATCTAACGGCGACCAAG	This study	1
R_spm_001	AACGGCGAAGAGACAATGACAAC	This study	1
F_vim_03	GTGTTTGGTCGCATATCGCAAC	This study	1
R_vim_03	GCTGTATCAATCAAAAGCAACTCATC	This study	1
F_cmy_01	CAGGTGCTCTTCAACAAG	This study	2
R_cmy_01	CGCCCTCTTTCTTTCAAC	This study	2
F_IS51_01	GTCATAGTATTCGTCGTTAGA	This study	2
R_IS51_01	GTAAGAGTGCTTTAATGTTCATA	This study	2
F_ndm_01	CGATCAAACCGTTGGAAG	This study	2
R_ndm_01	AAGGAAAACTTGATGGAATTG	This study	2
F_oxa24_02	ACTTTAGGTGAGGCAATG	This study	2
R_oxa24_02	TAACTTCTTGTACTGGTGTAA	This study	2
F_oxa27_001	ATATTTTACTTGCTATGTGGTTGCTTCTC	This study	2
R_oxa27_001	TCTCCAATCCGATCAGGGCATTC	This study	2
F_oxa58_02	GACAATTACACCTATACAAGAAG	This study	2
R_oxa58_02	CGCTCTACATACAACATCTC	This study	2
F_sfc_01	CCTGGTGATGATAGAGATAC	This study	2
R_sfc_01	ATAATCGTTGGCTGTACC	This study	2
F_sim_01	CTGCTGGGATAGAGTGGCTTAATAC	This study	2
R_sim_01	TCAATAGTGATGCGTCTCCGATTTC	This study	2

**Table 3:** Control strains.

Gene	Bacterium	Ct (50 ng/µl)	T(m)	PCR reaction
GES-1	*K. pneumoniae*	23	84	PCR1
GIM-1	*P. aeruginosa*	16	80	PCR1
IMI-2	*E. cloacae*	14	78	PCR1
IMP-15	*P. aeruginosa*	15	77	PCR1
KPC-2	*K. pneumoniae*	17	87	PCR1
OXA-48	*E. coli*	15	75	PCR1
SME	*S. marcescens*	11	77	PCR1
SPM-1	*P. aeruginosa*	16	80	PCR1
VIM-1	*K. pneumoniae*	17	81	PCR1
CMY-1/10	*E.coli* *	16	88	PCR2
ISaba1-OXA-51- family	*Acinetob. spp*	19	72	PCR2
NDM-1	*K. pneumoniae*	18	87	PCR2
OXA-23- family	*Acinetob. spp*	22	78	PCR2
OXA-24/40- family	*Acinetob. spp*	17	79	PCR2
OXA-58	*E.coli* *	15	76	PCR2
SFC-1	*E.coli* *	16	81	PCR2
SIM-1	*Acinetob. spp*	21	80	PCR2

* =  gene construct containing the partial, non-functional resistance gene in *E. coli* plasmid.

The PCR was run as a preformed oligonucleotide mixture with master mixture and template to avoid quality variations between the runs. A new oligonucleotide mixture was always tested with all the panel targets with set expected 19–25 Cq range in qPCR depending on the target (Table 3). The oligonucleotide mixture was stored in stock concentrations in small aliquots, and a working dilution was formed for short term usage only. In addition, each PCR run including a representative negative and positive control for the given multiplex: KPC for multiplex 1 and NDM for multiplex 2. An acceptance range for positive controls (target +/−3 Cq) was implemented to accept test series.

All positive isolates were confirmed by further analysing by an independent, conventional PCR and by sequencing the carbapenemase gene. Primers used in sequencing are presented in Table 4. Reaction included 2,5 mM dNTP 1,6 µl, HotStarTaq polymerase (Qiagen, Helsinki, Finland), 0,1 µl, Polymerase Buffer 10×2 µl, primer F and R 1 µl each, H_2_0 13,3 µl and 1 µl template making a total of 20 µl reaction volume. Amplification was performed as follows: initial denaturation 95°C 15 min, 35 cycles with denaturation 94°C 30 sec, variable annealing temperature 55/60/62°C 30 sec depending on the carbapenemase gene to be amplified, extension 72°C 10 min, final extension 72°C 10 min (DNA Engine Tetrad 2, Peltier Thermal Cycler, BioRad, CA, USA).

**Table 4:** Primers used for sequencing of resistance genes by polymerase chain reaction (PCR).

Gene	Primer	Sequence (5′- 3′)	Size (bp)	T (m)	Reference
**CMY**	F_cmy_s1	TAAGATACTTCGGATGAGGAG	695	60	
	R_cmy_s1	GCATCTTCTCGGATGAATC			This study
	GES-C	GTTTTGCAATGTGCTCAACG	371	60	
**GES**	GES-D	TGCCATAGCAATAGGCGTAG			[25]
	GIM-1F	AGAACCTTGACCGAACGCAG	748	60	
**GIM**	GIM-1R	ACTCATGACTCCTCACGAGG			[25]
	IMI-A	ATAGCCATCCTTGTTTAGCTC	818	55	
**IMI**	IMI-B	TCTGCGATTACTTTATCCTC			[25]
	F_IMP-1	TGAGCAAGTTATCTGTATTC	740	55	
**IMP**	R_IMP-1	TTAGTTGCTTGGTTTTGATG			[25]
	F_IMP-2	GGCAGTCGCCCTAAAACAAA	737	55	
**IMP**	R_IMP-2	TAGTTACTTGGCTGTGATGG			[25]
**ISaba1/OXA-51**	F_IS51_01	GTCATAGTATTCGTCGTTAGA	301	60	
	R_oxa51_001	GCACGAGCAAGATCATTACCATAGC			This study
	F_KPC	ATGTCACTGTATCGCCGTCT	893	55	
**KPC**	R_KPC	TTTTCAGAGCCTTACTGCCC			[25]
**NDM**	F_ndm_s1	GACAACGCATTGGCATAAG	447	60	
	R_ndm_s1	AAAGGAAAACTTGATGGAATTG			This study
**OXA-23 family**	F_oxa23_s1	GTGTCATAGTATTCGTCGTTAG	592	60	
	R_oxa23_s1	TATCAACCTGCTGTCCAAT			This study
**OXA-24 family**	F_oxa25_s1	ATTAGGGCTTGAGTGGAAA	521	60	
	R_oxa25_s1	TTGTATGATTGTCAACTGCTAT			This study
	OXA-48A	TTGGTGGCATCGATTATCGG	744	62	
**OXA-48**	OXA-48B	GAGCACTTCTTTTGTGATGGC			[25]
**SFC**	F_sfc_s1	CTCATTCTCCTGTGACTGA	351	60	
	R_sfc_s1	TTGCTCCTCCTGTTGTATT			This study
	SIM1-F	TACAAGGGATTCGGCATCG	571	60	
**SIM**	SIM1-R	TAATGGCCTGTTCCCATGTG			[25]
	F_sme_s1	AAGGCTCAGGTATGACATT	410	60	
**SME**	R_sme_s1	GGCATAATCATTCGCAGTA			This study
	SPM-1F	CCTACAATCTAACGGCGACC	650	55	
**SPM**	SPM-1R	TCGCCGTGTCCAGGTATAAC			[25]
	F_VIM-1	TTATGGAGCAGCAACCGATGT	920	60	
**VIM**	R_VIM-1	CAAAAGTCCCGCTCCAACGA			[25]
	F_VIM-2	AAAGTTATGCCGCACTCACC	865	60	
**VIM**	R_VIM-2	TGCAACTTCATGTTATGCCG			[25]

### Rep-PCR

DNA was extracted from colonies on CLED plates using the UltraClean microbial DNA isolation kit (Mo Bio Laboratories, Solona Beach, CA, USA) and diluted to 35 ng/µl. The DNA was amplified using the DiversiLab *Acinetobacter* kit (Bacterial Barcodes, Inc. cat no DL-AB01, Athens, GA, USA) for DNA fingerprinting following the manufacturer's instructions. PCR was run on preheated thermal cycler (DNA Engine Tetrad 2, Peltier Thermal Cycler BioRad, Hercules, CA, USA) using the parameters according to manufacturer's recommendations. The kit specific positive and negative controls were run with each reaction set for the validation of amplification. The rep-PCR products were detected and the amplicons were separated using microfluidics lab-on-a-chip technology and analysed using the DiversiLab system (Bacterial Barcodes, Inc.). Further analysis was performed with the web-based DiversiLab software (version 3.4) using the band-based modified Kullback-Leibler distance for the calculation of percent similarities. The manufacturer provides guidelines for strain-level discrimination; similarity more than 97% is considered as indistinguishable (no differences in fingerprints), similarity more than 95% as similar (1-2 band difference in fingerprints) and similarity less than 95% as different. In this study optimal cut-off for clustering was 95%.

### Ethics statement

The bacterial isolates analyzed in this study belong to the microbiological collections of HUSLAB (Laboratory of Helsinki University Central Hospital) and were obtained as part of routine clinical care in the past. Furthermore, all patient identifiers had been previously removed and data were analyzed anonymously. As the isolates were not clinical samples in the legal sense, no written or verbal consent was needed.

## Results

### Characterization of carbapenemase genes with *A. baumannii*

**Figure 1:**
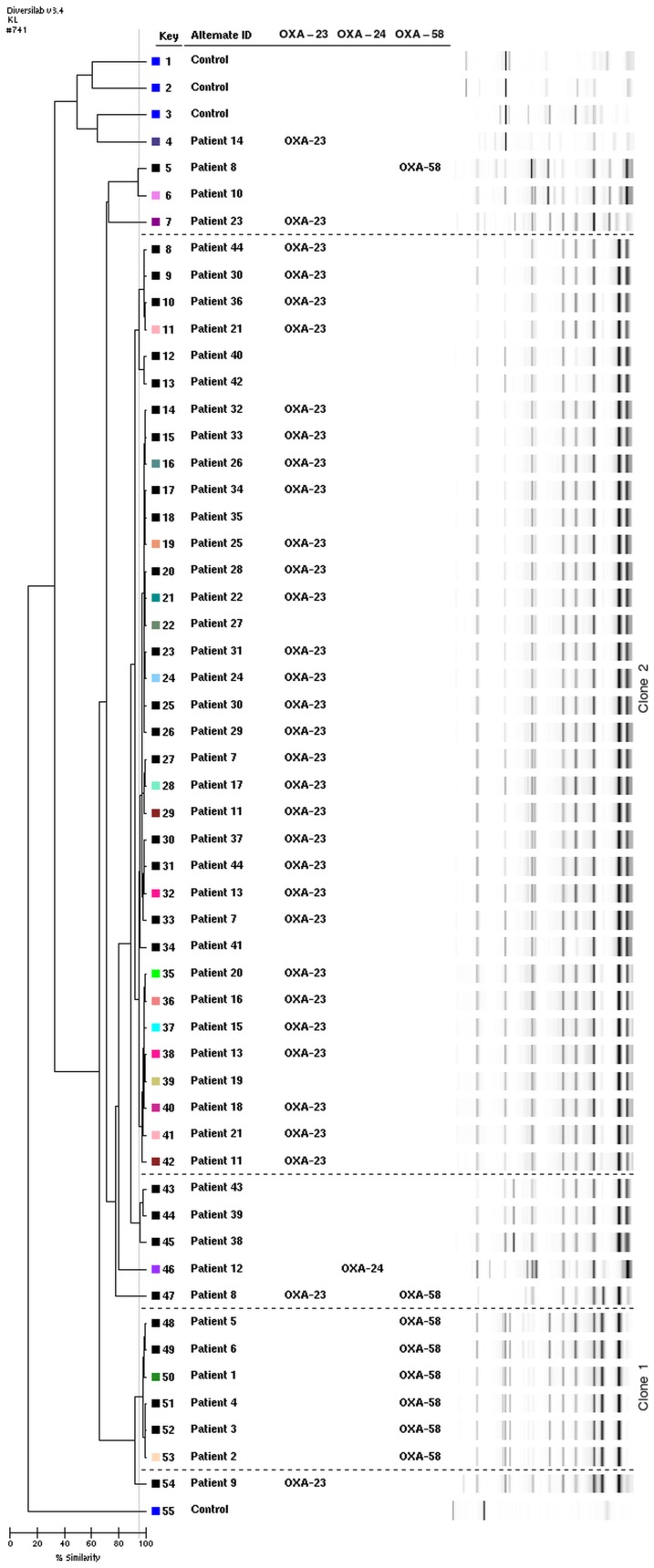
DiversiLab analysis. Dendogram and computer-generated image of rep-PCR banding patterns showing clustering between oxacillinase genes; OXA-23-like, OXA-24-like and OXA-58.

All the strains were analysed for 17 carbapenemase gene groups using the new assay. Among these *A. baumannii* isolates the most prevalent gene was OXA-23-like (*bla*_OXA-23-like_). In addition we also found eight OXA 58 (*bla*_OXA-58_) genes and one OXA-24-like (*bla*_OXA-24-like_) gene (Figure 1). No other carbapenemase genes, including genes for KPC, VIM, IMP, GES-1/-10, OXA-48, NDM, GIM-1, SPM-1, IMI/NMC-A, SME, CMY-10, SFC-1, and SIM-1, were detected. The IS*Aba*I-OXA-51-like junction PCR was negative in all strains, as well (data not shown).

### Temporal variation of prevalent, endemic *A. baumannii* clones

**Figure 2:**
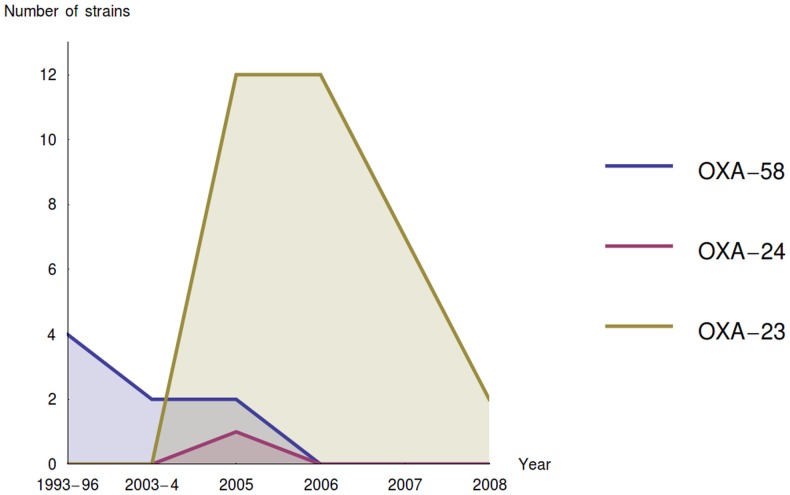
Time-dependent distribution of acquired oxacillinase genes; OXA-23-like, OXA-24-like and OXA-58.

A time dependent clonal variation among the analysed *A. baumannii* was observed. A predominant clone was detected during the follow-up period, typically lasting a few years, which was then substituted by a new clone (Figure 2). Briefly, first a few isolates, harbouring a mobile element with OXA-58 gene, appeared 1993–1996 and 2003–2006 (Clone 1, Figure 1), which was not detected in the following years, followed by a clone harbouring a mobile OXA-23-like gene (Clone 2, Figure 1). The results were consistent with DiversiLab typing, and characteristic antibiotic susceptibility profile associated with the OXA clones analyzed. Only five out of 55 species having OXA-23/-58 gene displayed a different rep-PCR profile. Based on rep-PCR analysis, two predominant clones were detected. One isolate having OXA-24-like gene was unique in DiversiLab analysis, as well. As expected, all the control isolates from patient with no known connection were unique in their rep-PCR profiles.

### Association of antibiotic susceptibility with clonality and carbapenemase gene profile

In our study, OXA-58 isolates had lower MIC-values for to meropenem than OXA-23-like positive isolates that systematically had higher MIC-values (Table 5). The isolates with non-acquired OXA-gene, displayed a marked variation and they included also some carbapenem resistant isolates. The control isolates (Figure 1) consisted of *Acinetobacter* spp not harbouring any of the OXA genes analyzed. These isolates were all carbapenem susceptible (Table 5).

**Table 5:** MIC distributions for 55 *Acinetobacter* isolates.

	Cumulative percentage of isolates inhibited at MIC (mg/l) value of:										
Drug	≤0.5	≤1	≤2	≤4	≤8	≤16	≤32	≤64	≤128	≤256	Isolate
**MP**	0,0	0,0	12,5	75,0	75,0	87,5	100,0				OXA-58
**IP**	0,0	0,0	0,0	0,0	12,5	37,5	100,0				OXA-58
**RI**	0,0	0,0	0,0	75,0	75,0	75,0	100,0				OXA-58
**AB**	0,0	0,0	12,5	37,5	50,0	50,0	87,5	87,5	87,5	100,0	OXA-58
**TGC**	0,0	12,5	25,0	87,5	87,5	87,5	87,5	100,0			OXA-58
**CO**	100,0										OXA-58
**MP**	0,0	0,0	0,0	0,0	0,0	0,0	100,0				OXA-23
**IP**	0,0	0,0	0,0	0,0	0,0	3,0	100,0				OXA-23
**RI**	0,0	0,0	0,0	85,0	88,0	88,0	100,0				OXA-23
**AB**	0,0	0,0	0,0	6,0	12,0	74,0	89,0	95,0	95,0	100,0	OXA-23
**TGC**	0,0	6,0	12,0	94,0	97,0	100,0					OXA-23
**CO**	100,0										OXA-23
**MP**	0,0	0,0	0,0	0,0	0,0	0,0	100,0				OXA-24
**IP**	0,0	0,0	0,0	0,0	0,0	0,0	100,0				OXA-24
**RI**	0,0	0,0	0,0	100,0							OXA-24
**AB**	0,0	0,0	0,0	0,0	0,0	0,0	0,0	0,0	0,0	100,0	OXA-24
**TGC**	0,0	0,0	100,0								OXA-24
**CO**	100,0										OXA-24
**MP**	20,0	40,0	50,0	50,0	50,0	80,0	100,0				non OXA
**IP**	10,0	50,0	50,0	50,0	50,0	50,0	100,0				non OXA
**RI**	0,0	0,0	0,0	30,0	90,0	90,0	100,0				non OXA
**AB**	0,0	0,0	0,0	0,0	20,0	90,0	100,0				non OXA
**TGC**	0,0	0,0	0,0	70,0	100,0						non OXA
**CO**	100,0										non OXA
**MP**	75,0	100,0									Control
**IP**	100,0										Control
**RI**	0,0	25,0	50,0	50,0	75,0	100,0					Control
**AB**	0,0	75,0	75,0	75,0	75,0	75,0	75,0	75,0	100,0		Control
**TGC**	50,0	100,0									Control
**CO**	100,0										Control

MP, meropenem; IP, imipenem; RI, rifampicin; AB, ampicillin+sulbactam; TCG, tigecycline; CO, colistin.

## Discussion

The carbapenemase producing multi-resistant gram negative rods are probably the most important challenge for hospital hygiene at the moment [13], [19]. The great variety of underlying mechanisms, in contrast to simple *mecA* or *mecC* in MRSA, possesses a significant challenge to clinical screening process. Phenotypes are highly variable and many overlapping other resistance mechanisms complicate any simple screening approach. A straight-forward, economical method suitable for routine clinical diagnostics has not been available yet. In this paper we demonstrate the good performance of a new multiplex real-time PCR assay, detecting most important carbapenemases based on melting curve analysis, by applying it to an epidemiologically important set of clinical *A. baumannii* isolates. In a striking contrast to carbapenemase producing *Enterobacteriaceae*, which were first detected in Finland 2008 [14], the carbapenem resistant *A. baumannii* were detected in Finland already three decades ago. This study highlights the emergence of carbapenem-resistant *A. baumannii* isolates carrying the *bla*_OXA-23-like_ gene (Clone 1), which replaced the *bla*_OXA-58_ gene (Clone 2) in three years (Figure 2). These major clones might have been endemic.

The new carbapenemase detection assay was initially developed to detect carbapenemase producing *Enterobacteriaceae* isolates, but it also appeared to be a useful tool for *P. aeruginosa* and *A. baumannii*. After three years of clinical use, it has been proved to be sensitive and highly specific screening assay among more than 700 hundred isolates with reduced carbapenem susceptibility analysed to date [14]. One of the major problems related to molecular detection of many antibiotic resistance genes is the appearance of new genomic variants. For example, the variable regions of *bla*_OXA-181_ are up to 9% different from *bla*_OXA-48_ [20]. The new variants may not be detectable with the existing systems. To minimize the risk for false negative results, the primers were designed at conserved gene regions to achieve optimal amplification of all the current and forthcoming sub-variants. The SYBR Green chemistry was preferred to avoid false negative results due to minor mutations in the probe sequence. The probe based assays are often sensitive to just 1–2 mutations in probe sequence, whereas primers are usually less sensitive to minor target mutations. These design features were considered relevant to achieve a high exclusion power of clinically relevant, acquired carbapenemase genes among carbapenem resistant strains.

*A. baumannii* is a nosocomial pathogen, and epidemiological tools are important to develop effective strategies for better monitoring of MDRAB clinical isolates [21]. In this study we used rep-PCR because the method is suitable for comparison of isolate genetic profiles using standardized and automated format [22]. This method has previously demonstrated good discrimination ability of *A. baumannii* isolates [23], [24]. We found two major clones with DiversiLab (Clone 1 and 2, Figure 1.) harbouring most of the isolates with *bla*_OXA-23-like_ and *bla*_OXA-58_ genes. There were only few exceptions. The cases were mostly from departments of treating patients with severe burn trauma, or intensive care units.

In this study, a good correlation between the carbapenemase gene and DiversiLab typing suggested that they both could be effectively applied for epidemiological screening of *A. baumannii* species. The new carbapenemase gene screening assay has been in clinical use for more than three years, and it has been a highly suitable method for rapid unequivocal identification of isolates harbouring acquired carbapenemase genes among *Acinetobacter, Pseudomonas aeruginosa*, and *Enterobacteriaceae* species. This study suggests that the new molecular methods could be successfully applied in clinical diagnostics to monitor acquired carbapenemase genes, provided that they are user-friendly and cost-effective as well.

## Supporting Information

Table S1 **Acinetobacter isolate description.**
(DOCX)

Table S2 **Species included in analytical specificity testing.**
(DOCX)
