# Supplementary material for: Rapid Molecular Characterization of Acinetobacter baumannii Clones with rep-PCR and Evaluation of Carbapenemase Genes by New Multiplex PCR in Hospital District of Helsinki and Uusimaa
Source: PLoS One. 2014 Jan 21;9(1):e85854. doi: 10.1371/journal.pone.0085854 (PMC3897539; doi:10.1371/journal.pone.0085854)
Supplement: Table S1 — Acinetobacter isolate description. (DOCX) [file pone.0085854.s001.docx]

| **Table S1.** |  |  |  |
| --- | --- | --- | --- |
| Acinetobacter isolate description | | |  |
|  |  |  |  |
| **Patient** | **Isolation site** | **Hospital** | **Origin** |
| Patient 1 | burn wound | 1 | n/a |
| Patient 2 | burn wound | 1 | n/a |
| Patient 3 | blood | 1 | n/a |
| Patient 4 | burn wound | 1 | n/a |
| Patient 5 | incision wound | 9 | n/a |
| Patient 6 | trachea | 9 | n/a |
| Patient 7 | wound | 1 | n/a |
| Patient 7 | incision wound | 1 | n/a |
| Patient 8 | wound | 2 | Asia |
| Patient 8 | trachea | 2 | Asia |
| Patient 9 | trachea | 2 | n/a |
| Patient 10 | blood | 1 | Russia |
| Patient 11 | CV catheter | 5 | n/a |
| Patient 11 | blood | 5 | Thailand |
| Patient 12 | trachea | 1 | Spain |
| Patient 13 | urinary catheter | 6 | n/a |
| Patient 13 | urine | 4 | n/a |
| Patient 14 | trachea | 1 | n/a |
| Patient 15 | trachea | 1 | n/a |
| Patient 16 | trachea | 1 | n/a |
| Patient 17 | incision wound | 1 | n/a |
| Patient 18 | incision wound | 1 | n/a |
| Patient 19 | incision wound | 1 | n/a |
| Patient 20 | incision wound | 1 | n/a |
| Patient 21 | dialysis catheter | 2 | n/a |
| Patient 21 | stool | 2 | n/a |
| Patient 22 | blood | 2 | n/a |
| Patient 23 | trachea | 8 | n/a |
| Patient 24 | wound | 7 | n/a |
| Patient 25 | stool | 2 | n/a |
| Patient 26 | stool | 2 | n/a |
| Patient 27 | stool | 1 | n/a |
| Patient 28 | stool | 1 | n/a |
| Patient 29 | stool | 1 | n/a |
| Patient 30 | wound | 1 | n/a |
| Patient 30 | incision wound | 1 | n/a |
| Patient 31 | incision wound | 1 | n/a |
| Patient 32 | wound | 2 | n/a |
| Patient 33 | incision wound | 1 | n/a |
| Patient 34 | incision wound | 1 | n/a |
| Patient 35 | incision wound | 1 | n/a |
| Patient 36 | wound | 6 | n/a |
| Patient 37 | incision wound | 1 | n/a |
| Patient 38 | incision wound | 1 | n/a |
| Patient 39 | wound | 5 | n/a |
| Patient 40 | wound | 2 | n/a |
| Patient 41 | CV catheter | 2 | n/a |
| Patient 42 | urinary catheter | 2 | n/a |
| Patient 43 | urinary catheter | 5 | n/a |
| Patient 44 | urine | 3 | n/a |
| Patient 44 | wound | 3 | n/a |
