# Supplementary material for: Rapid Molecular Characterization of Acinetobacter baumannii Clones with rep-PCR and Evaluation of Carbapenemase Genes by New Multiplex PCR in Hospital District of Helsinki and Uusimaa
Source: PLoS One. 2014 Jan 21;9(1):e85854. doi: 10.1371/journal.pone.0085854 (PMC3897539; doi:10.1371/journal.pone.0085854)
Supplement: Table S2 — Species included in analytical specificity testing. (DOCX) [file pone.0085854.s002.docx]

| **Table S2.** |
| --- |
| Species included in analytical specificity testing |
|  |
| **Species** |
| *Alcaligenes faecalis* |
| *Citrobacter amalonaticus* |
| *Citrobacter amalonaticus* |
| *Citrobacter freundii* |
| *Citrobacter freundii* |
| *Citrobacter freundii* |
| *Enterobacter aerogenes* |
| *Enterobacter aerogenes* |
| *Enterobacter aerogenes* |
| *Enterobacter cloacae* |
| *Enterobacter cloacae* |
| *Escherichia coli* |
| *Escherichia coli* |
| *Escherichia coli* |
| *Hafnia alvei* |
| *Hafnia alvei* |
| *Hafnia alvei* |
| *Klebsiella oxytoca* |
| *Klebsiella oxytoca* |
| *Klebsiella oxytoca* |
| *Klebsiella oxytoca* |
| *Klebsiella pneumoniae* |
| *Klebsiella pneumoniae* |
| *Klebsiella pneumoniae* |
| *Morganella morganii* |
| *Morganella morganii* |
| *Morganella morganii* |
| *Proteus mirabilis* |
| *Proteus mirabilis* |
| *Proteus mirabilis* |
| *Proteus vulgaris* |
| *Proteus vulgaris* |
| *Proteus vulgaris* |
| *Pseudomonas acidovorans* |
| *Pseudomonas aeruginosa* |
| *Pseudomonas aeruginosa* |
| *Pseudomonas diminuta* |
| *Pseudomonas fluorescens* |
| *Pseudomonas fluorescens* |
| *Pseudomonas fluorescens* |
| *Pseudomonas putida* |
| *Pseudomonas spp.* |
| *Pseudomonas spp.* |
| *Pseudomonas spp.* |
| *Pseudomonas stutzeri* |
| *Pseudomonas stutzeri* |
| *Salmonella choleraisuis* |
| *Salmonella dublini* |
| *Salmonella enteritidis* |
| *Salmonella paratyphi A* |
| *Salmonella stanley* |
| *Salmonella typhi* |
| *Salmonella typhimurium* |
| *Salmonella virchow* |
| *Serratia marcescens* |
| *Serratia marcescens* |
| *Serratia marcescens* |
| *Stenotrophomonas maltophilia* |
